# Fluid Retention Caused by Rosiglitazone Is Related to Increases in AQP2 and *α*ENaC Membrane Expression

**DOI:** 10.1155/2017/8130968

**Published:** 2017-11-02

**Authors:** Jinghua Xu, Mingyue Pan, Xiaoli Wang, Lishi Xu, Lanfang Li, Cheng Xu

**Affiliations:** Department of Physiology, School of Life Science and Biopharmaceutics, Shenyang Pharmaceutical University, 103 Wenhua Road, Shenyang 110016, China

## Abstract

Peroxisome proliferator activated receptor-*γ* (PPAR*γ*) is a ligand-activated transcription factor of the nuclear hormone receptor superfamily. The decreased phosphorylation of PPAR*γ* due to rosiglitazone (ROS) is the main reason for the increased insulin sensitivity caused by this antidiabetic drug. However, there is no clear evidence whether the nuclear translocation of p-PPAR*γ* stimulated by ROS is related to fluid retention. It is also unclear whether the translocation of p-PPAR*γ* is associated with the change of aquaporin-2 (AQP2) and epithelial sodium channel *α* subunit (*α*ENaC) in membranes, cytoplasm, and nucleus. Our experiments indicate that ROS significantly downregulates nuclear p-PPAR*γ* and increases membrane AQP2 and *α*ENaC; however, SR1664 (a nonagonist PPAR*γ* ligand) reduces p-PPAR*γ* and has no effect on AQP2 and *α*ENaC. Therefore, we conclude that in vitro the fluid retention caused by ROS is associated with the increases in membrane *α*ENaC and AQP2 but has little relevance to the phosphorylation of PPAR*γ*.

## 1. Introduction

Rosiglitazone (ROS), a classic clinical oral antidiabetic drug, is a PPAR*γ* agonist; studies have shown that the decreased phosphorylation of PPAR*γ* due to ROS is the main reason for the increase in insulin sensitivity caused by this drug [1]. However, long-term clinical observations have revealed that ROS has the side effect of fluid retention, which leads to heart failure [2, 3]. SR1664, a novel compound developed by the Scripps Institute in the United States and other institutions, is a PPAR*γ* ligand that activates PPAR*γ* to suppress PPAR*γ* Ser^273^ phosphorylation, leading to enhanced insulin sensitivity, thereby playing a role in treating type 2 diabetes [4]. However, in vivo experiments showed that SR1664 can increase insulin sensitivity and does not cause fluid retention [5].

Substantial research showed that fluid retention is closely related to AQP2 and *α*ENaC proteins [6, 7], and in vitro experiments indicated that the expression of AQP2 and *α*ENaC is upregulated after PPAR*γ* agonist treatment [8, 9]. Nevertheless, the mechanism of ROS on membrane, cytoplasmic, and nuclear AQP2 and *α*ENaC has yet to be clarified. In this study, we investigated the effects of ROS, SR1664, and TNF*α* (increased phosphorylation of PPAR*γ*) on p-PPAR*γ*, AQP2, and *α*ENaC in HEK293 and mIMCD-3 cells, being then coincubated with PPAR*γ* antagonist GW9662; results showed that the effects disappeared. So we concluded that in vitro the decrease of PPAR*γ* phosphorylation has little relationship with fluid retention, and the fluid retention induced by ROS is mainly related to the increase of membranes AQP2 and *α*ENaC.

## 2. Materials and Methods

### 2.1. Chemicals and Reagents

Reagents were purchased from the following sources: rosiglitazone and GW9662 (Sigma, St Louis, MO); RPMI-1640 medium (GIBCO, Invitrogen) and fetal bovine serum (FBS; Shenyang Huibai Biotechnology Co., Ltd.); 3-(4,5-dimethylthiazol-2-yl)-2,5-diphenyl tetrazolium (MTT; Biosharp); and ECL Western Blotting Detection Reagent (Bio-Rad, Hercules, CA, USA). Antibodies were selected using polyclonal antibody, PPAR*γ* (Santa Cruz, CA, USA), p-PPAR*γ* (Proteintech, CA, USA), *α*ENaC (Santa Cruz, USA), AQP2 (Santa Cruz, USA), and *β*-tubulin (Santa Cruz, USA); FITC Alexa 488-conjugated goat anti-rabbit secondary antibodies were from Santa Cruz Biotechnology (USA); and horseradish peroxidase-conjugated anti-rabbit secondary antibodies were from Proteintech (USA).

### 2.2. Cell Culture and Administration

Mouse kidney inner medullary collecting duct (mIMCD) cells (Shanghai Bogu Biological Technology Co., Ltd.) and human embryo kidney (HEK293) cells (ATCC, Manassas, VA, USA) were routinely cultured in RPMI-1640 medium supplemented with 10% FBS and antibiotics (Serva & AMRESCO), in a humidified chamber containing 5% CO_2_ at 37°C. Combined administration regimen is as follows: when the cells reached 80%–90% confluence, GW9662 (5 *μ*M) was incubated for 6 h before the addition of ROS (1, 10 *μ*M) or SR1664 (1, 10 *μ*M). After 24 h, cells were collected for extraction of total, cytoplasmic, nucleus, or membrane protein, respectively.

### 2.3. Cell Viability Experiment

For the MTT assay, cells were seeded at 6 × 10^3^ cells per well onto 96-well culture plates and allowed to grow for 24 h after treatment with various concentrations of ROS, SR1664, GW9662, TNF*α*, or ROS and SR1664 combined with GW9662. After removing the medium, MTT solution (5 mg/ml in PBS) was added and incubated for 4 h and the resulting formazan was solubilized with DMSO (150 *μ*l). The absorption was measured at 490 nm in a multifunctional enzyme marking instrument.

### 2.4. Preparation of Protein Samples

#### 2.4.1. Preparation of Total Protein

RIPA lysis buffer was added to the cell precipitations and resuspended. After 30 min of lysis at 4°C, the lysates were centrifuged at 12,000 g for 20 min, and the obtained supernatant was used as the total protein.

#### 2.4.2. Preparation of Cytoplasm and Nuclear Proteins

The reagents were dissolved at room temperature and put on the ice immediately. Then, 200 *μ*l cytoplasmic protein extraction reagent A (to which 1 mM PMSF had been added a few minutes previously) was added to 20-*μ*l cell precipitations. Five seconds' vortex was performed to ensure adequate resuspension, followed by incubation on ice for 10–15 min. Then, 10 *μ*M cytoplasmic protein extraction reagent B was added. Vortexing was again performed for 5 s, followed by incubation on ice for 1 min. Centrifugation was then performed at 12,000 g and 4°C for 5 min, and the obtained supernatant was used as cytoplasmic protein. Then, 50 *μ*l of PMSF-added nucleoprotein extraction reagent was added to the nuclear pellet, followed by vortexing for 15–30 s, to ensure complete suspension and dispersal. Then, after incubation on ice, vortexing for 30 s was performed every 1-2 minutes for 30 min. This was followed by vortexing at 12,000 g at 4°C for 10 min, from which the obtained supernatant was used to represent nuclear protein. The nuclear and cytoplasmic extracts were then analyzed for protein content using BCA assay.

#### 2.4.3. Preparation of Membrane Proteins

The cellular membrane fraction was prepared in accordance with the manufacturer's instructions. 1 ml membrane protein extraction reagent A with PMSF was added to 2–5 billion cells, for gentle and complete suspension, followed by incubation on ice for 10–15 min. Next, centrifuging was applied at 700 g for 10 min at 4°C. The supernatant obtained from this procedure was then centrifuged at 14,000 g for 30 min at 4°C to precipitate membrane fragments, with the obtained supernatant being used to represent cytoplasmic protein. The precipitate was also centrifuged at 14,000 g for 10 s at 4°C and exhausted the supernatant completely. Then, after the addition of 200 *μ*l of membrane protein extraction reagent B, vortexing was performed for 5 s for resuspension, followed by incubation on ice for 5–10 min. The previous steps were then repeated 1-2 times to extract the membrane protein completely. Subsequently, centrifugation was performed at 14,000 g for 5 min at 4°C, with the obtained supernatant being used as the membrane protein. The membrane extracts were then analyzed for protein content using BCA assay.

### 2.5. Western Blot Analysis

Cells were first washed with cold PBS three times and lysed in RIPA buffer. The BCA protein assay was used to determine the protein concentrations of the samples. Equal amounts (25 ug) of cellular proteins were loaded into each well and separated by 10% SDS-PAGE after denaturation with 5x loading buffer and then transferred onto PVDF membranes, incubated in 5% nonfat dry milk for 2 h on shaker at room temperature and then incubated with PPAR*γ* (1 : 500), p-PPAR*γ* (1 : 500), AQP2 (1 : 800), and *α*ENaC (1 : 800) antibodies, respectively; *β*-tublin (1 : 1000) was used as internal control. Finally, blots were also incubated with secondary antibody (1 : 5000) and visualized using enhanced ECL luminous fluid.

### 2.6. Immunocytochemistry

Cells in the logarithmic growth phase were collected and, following adjustment of cell suspension density to 1 × 10^5^ cells/ml after digestion, the cells were inoculated on coverslips with concentrations of drugs diluted with culture medium, cultured at 37°C for 24 h, and blocked for 30 min without light at room temperature [4% paraformaldehyde (PFA), 0.2% Triton X-100, and 5% BSA]. Overnight incubation with primary antibodies (diluted with 1% BSA) was performed at 4°C followed by 30 min of incubation with secondary antibodies (diluted with 1% BSA). Washing with PBS was then performed three times for 10 min after each step, along with exposure to 100 ng/ml Hoechst 33258 dye for 10 min, then washing again with PBS for 5 min three times. Finally, confocal microscopy was performed using a 60x oil objective on a Nikon C2-si laser-scanning confocal microscope, and images were manipulated using Photoshop software.

### 2.7. Data Analysis

All experiments were repeated at least three times independently, and data are expressed as mean ± s.e.m. Statistical analysis was performed using SPSS 17.0 by one-way analysis of variance (ANOVA). The LSD test was used to compare differences in the means between groups; if the variance is different, Dunnett's *t*-test was used. A value of *P* < 0.05 was considered to represent a significant difference, while *P* < 0.01 was considered to present a very significant difference.

## 3. Results

### 3.1. Cell Viability Analysis

**Figure 1:**
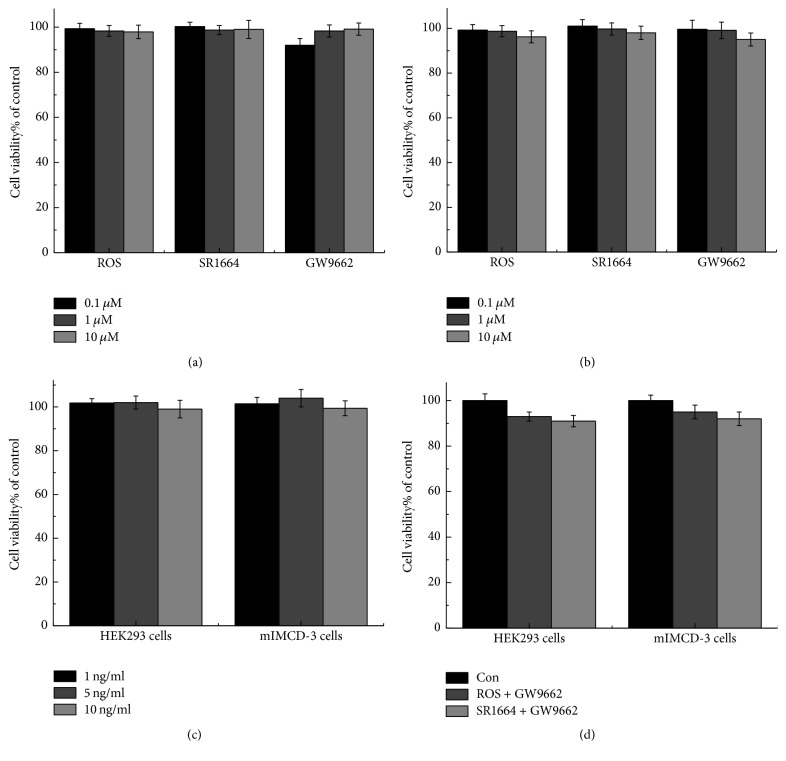
In vitro experiments on the cytotoxic effects of ROS, GW9662, SR1664, and TNF*α* in HEK293 and mIMCD-3 cells. (a) The effects of ROS, SR1664, and GW9662 (0.1, 1, and 10 *μ*M) in HEK293 cells. (b) The effects of ROS, SR1664, and GW9662 (0.1, 1, and 10 *μ*M) in mIMCD-3 cells. (c) The effects of TNF*α* (1, 5, and 10 ng/ml) in HEK293 and mIMCD-3 cells. (d) Coincubation with ROS (10 *μ*M) and GW9662 (5 *μ*M) and SR1664 (10 *μ*M) and GW9662 (5 *μ*M). The results show that the concentrations that we used in the experiments have no significant cytotoxic effects. Results are shown as mean ± s.e.m., *n* = 3.

First, to consider the cytotoxic effects of ROS, SR1664, GW9662, and TNF*α*, 3-(4,5-dimethylthiazol-2-y1)-2,5-diphenyltetrazolium bromide (MTT) experiments were used to determine the doses of these drugs and combination therapies. The administration of ROS, SR1664, and GW9662 at 0.1, 1, and 10 *μ*M (Figures 1(a) and 1(b)) and TNF*α* at 1, 5, and 10 ng/ml (Figure 1(c)) to HEK293 cells and mIMCD-3 cells showed no significant effects on cell viability. The administration of these agents in combination (Figure 1(d) also had no such effects.

### 3.2. Immunofluorescence Assay

**Figure 2:**
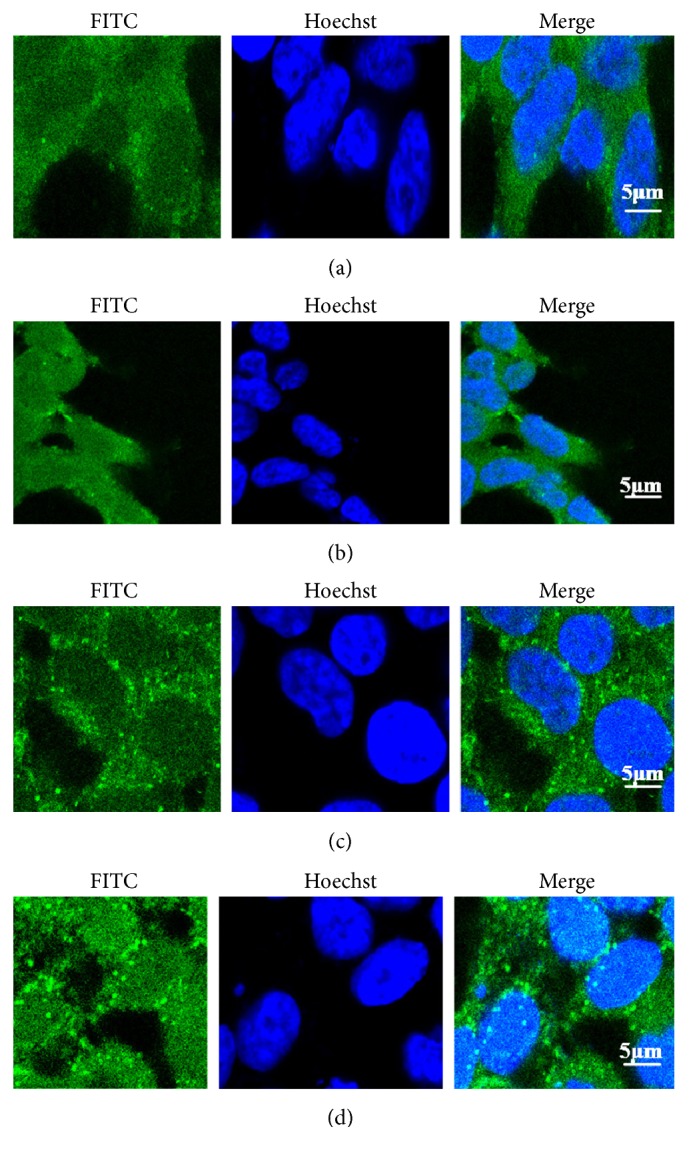
Altered localization of PPAR*γ* in response to rosiglitazone. (a, c) Control in HEK293 and mIMCD-3 cells, respectively. PPAR*γ* immunoreactivity following 24 h of culture without rosiglitazone; (b, d) PPAR*γ* immunoreactivity following 24 h of culture with rosiglitazone (10 *μ*M). Column 2 demonstrates nuclear staining (Hoechst; blue); column 3 illustrates PPAR*γ* immunoreactivity (localized using Alexa 488; green). Column 1 features overlay images. PPAR*γ* is redistributed to the nucleus in response to elevated rosiglitazone at 10 *μ*M.

Activation of nuclear receptor promotes the translocation of transcription factors from the cytoplasm to the nucleus to improve the transcriptional activity of transcription factor response element binding (CREB) protein. Therefore, a cell immunofluorescence experiment was used to detect the changes in the distribution of nuclear receptor PPAR*γ* after the administration of agonists, which was based on the binding of a green fluorescence in the cytoplasm and nucleus; Hoechst stains the nucleus and indicates the localization of PPAR*γ* in cells. The results are shown in Figure 2, indicating that, after ROS administration, green fluorescence in the cell nucleus increased compared with that in the blank group. Data analysis suggested that there was no significant difference in this activity in the cytoplasm, but it increased markedly in the nucleus.

### 3.3. Effects of ROS on PPAR*γ*, p-PPAR*γ*, AQP2, and *α*ENaC in HEK293 Cells

**Figure 3:**
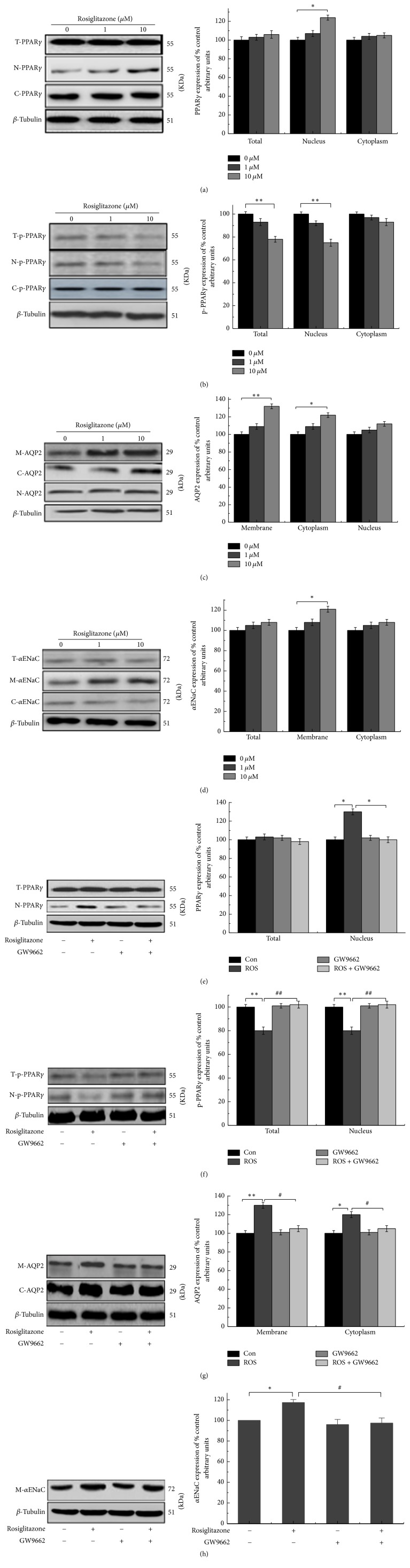
Effects of rosiglitazone and coincubation with GW9662 in HEK293 cells. (a–d) The effects of rosiglitazone on PPAR*γ*, p-PPAR*γ*, AQP2, and *α*ENaC. (e–h) The changes of PPAR*γ*, p-PPAR*γ*, AQP2, and *α*ENaC upon coincubation with GW9662 (10 *μ*M ROS + 5 *μ*M GW9662). The results show that rosiglitazone at 10 *μ*M can significantly decrease total and nuclear p-PPAR*γ* but critically upregulated nuclear PPAR*γ* and AQP2 and *α*ENaC membrane transposition. After coincubation with GW9662 (10 *μ*M ROS + 5 *μ*M GW9662), the effects of rosiglitazone on PPAR*γ*, p-PPAR*γ*, AQP2, and *α*ENaC disappeared. The results are shown as mean ± s.e.m., *n* = 3. ^*∗*/#^*P* < 0.05. ^*∗∗*/##^*P* < 0.01. *∗* means compared to control; # means compared to rosiglitazone.

To investigate the effects of ROS on PPAR*γ*, p-PPAR*γ*, AQP2, and *α*ENaC at different locations, total, nuclear, membrane, and cytoplasmic proteins were extracted. The data show that ROS at 10 *μ*M can significantly increase nuclear PPAR*γ* (N-PPAR*γ*) (Figure 3(a)) but downregulated total p-PPAR*γ* (T-p-PPAR*γ*) and nuclear p-PPAR*γ* (N-p-PPAR*γ*) and had no influence on cytoplasmic p-PPAR*γ* (C-p-PPAR*γ*) (Figure 3(b)). As for AQP2, membrane (M-AQP2) and cytoplasmic (C-AQP2) proteins levels were increased (Figure 3(c)), while, for *α*ENaC, the findings show that just membrane (M-*α*ENaC) translocation increased (Figure 3(d)). After coincubation with GW9662, the effects of ROS on PPAR*γ*, p-PPAR*γ*, AQP2, and *α*ENaC disappeared (Figures 3(e)–3(h)).

### 3.4. Effects of ROS on PPAR*γ*, p-PPAR*γ*, AQP2, and *α*ENaC in mIMCD-3 Cells

**Figure 4:**
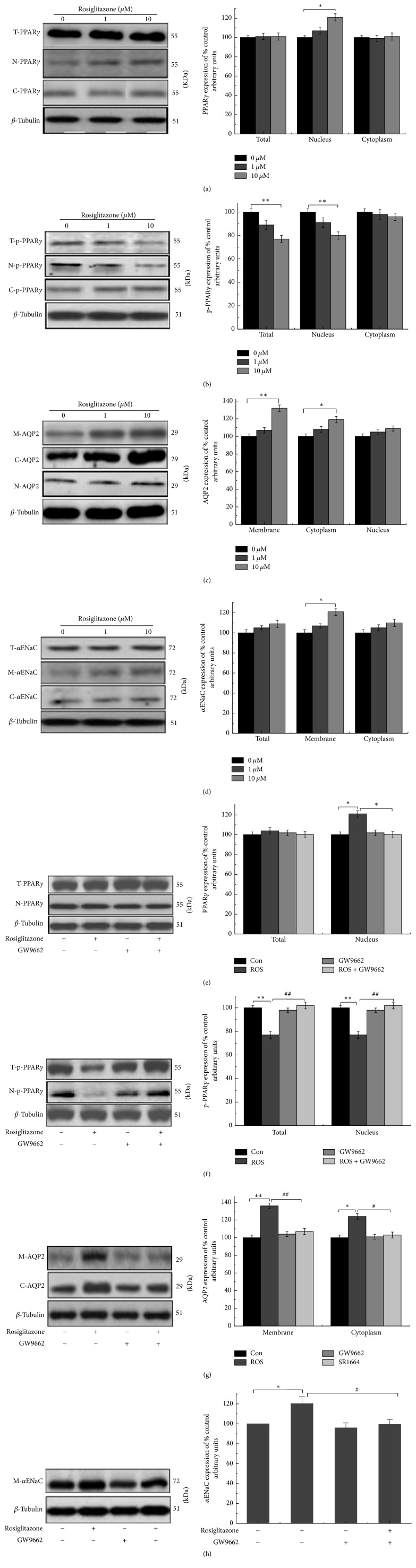
Effects of rosiglitazone and coincubation with GW9662 in mIMCD-3 cells. (a–d) The effects of rosiglitazone on PPAR*γ*, p-PPAR*γ*, AQP2, and *α*ENaC. (e–h) The changes of PPAR*γ*, p-PPAR*γ*, AQP2, and *α*ENaC upon coincubation with GW9662 (10 *μ*M ROS + 5 *μ*M GW9662). The results show that rosiglitazone at 10 *μ*M can significantly decrease total and nuclear p-PPAR*γ* but critically upregulated nuclear PPAR*γ* and AQP2 and *α*ENaC membrane transpositions. After coincubation with GW9662 (10 *μ*M ROS + 5 *μ*M GW9662), the effects of rosiglitazone on PPAR*γ*, p-PPAR*γ*, AQP2, and *α*ENaC disappeared. The results are shown as mean ± s.e.m., *n* = 3. ^*∗*/#^*P* < 0.05. ^*∗∗*/##^*P* < 0.01. *∗* means compared to control; # means compared to rosiglitazone.

To validate the above findings in HEK293 cells, the same experiments were conducted in mIMCD-3 cells. The data indicate that ROS (10 *μ*M) also critically increased nuclear PPAR*γ* (Figure 4(a)), downregulated total and nuclear p-PPAR*γ* in mIMCD-3 cells (Figure 4(b)), and increased the expression of AQP2 in cytoplasm and membrane (Figure 4(c)), as well as membrane expression of *α*ENaC (Figure 4(d)). Upon coincubation with GW9662, these effects disappeared (Figure 4(e)–4(h)). The results are consistent with those in HEK293 cells.

### 3.5. Effects of SR1664 and TNF*α* on PPAR*γ*, p-PPAR*γ*, AQP2, and *α*ENaC in mIMCD-3 Cells

**Figure 5:**
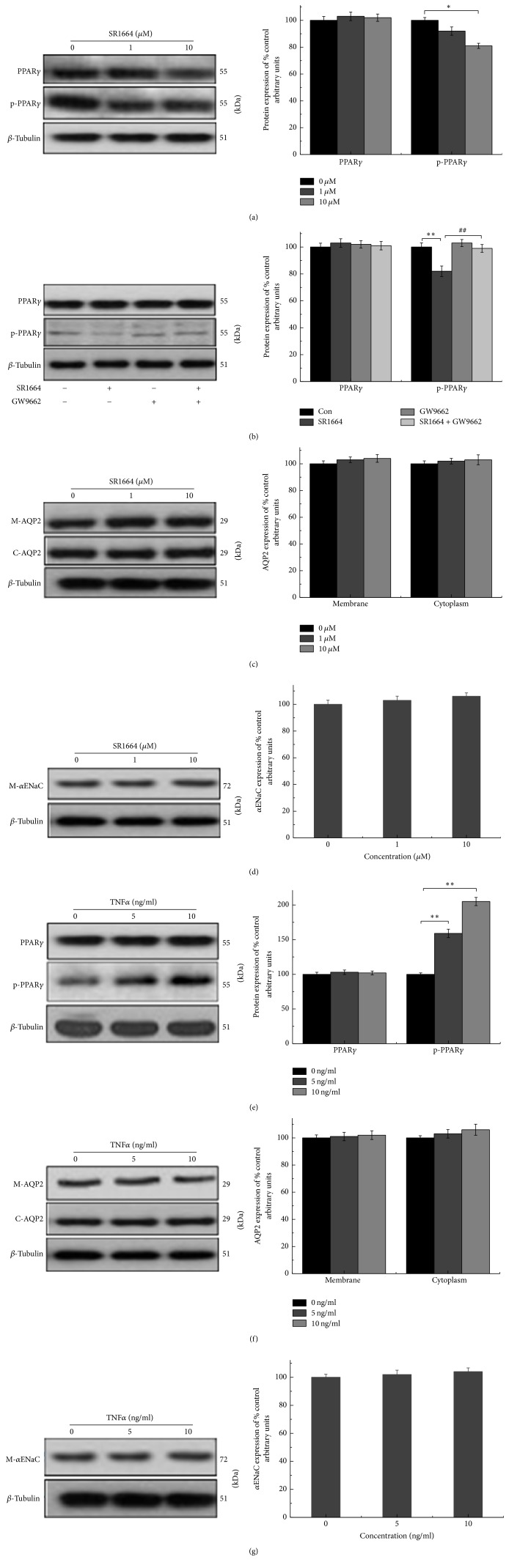
Effects of SR1664 and TNF*α* on PPAR*γ*, p-PPAR*γ*, AQP2, and *α*ENaC in mIMCD-3 cells. (a, c, d) The effects of SR1664 on PPAR*γ*, p-PPAR*γ*, AQP2, and *α*ENaC. (b) The changes of PPAR*γ* and p-PPAR*γ* upon coincubation with GW9662 (10 *μ*M SR1664 + 5 *μ*M GW9662). (e–g) The effects of TNF*α* on PPAR*γ*, p-PPAR*γ*, AQP2, and *α*ENaC. The results show that SR1664 at 10 *μ*M can significantly inhibit p-PPAR*γ*; TNF*α* even 5 ng/ml can critically increase p-PPAR*γ*, while there was no significant difference in the expression of AQP2 in cell membrane and cytoplasm and *α*ENaC in membrane. After coincubation with GW9662 (10 *μ*M SR1664 + 5 *μ*M GW9662), the effect of SR1664 on p-PPAR*γ* disappeared. The results are shown as mean ± s.e.m., *n* = 3. ^*∗*^*P* < 0.05. ^*∗∗*/##^*P* < 0.01. *∗* means compared to control; # means compared to SR1664.

SR1664 is a new type of PPAR*γ* ligand that blocks the cyclin-dependent kinase 5- (Cdk5-) mediated phosphorylation of PPAR*γ*. Research has shown that it improves insulin sensitivity by lowering glucose and has no side effects, as is the case for ROS. To confirm these effects, we detected PPAR*γ*, p-PPAR*γ*, AQP2, and *α*ENaC proteins in mIMCD-3 cells. The data suggest that SR1664 (10 *μ*M) can dramatically limit the p-PPAR*γ* (Figure 5(a)); upon coincubation with GW9662, the effects on p-PPAR*γ* disappeared (Figure 5(b)). Nevertheless, the expression of AQP2 in the cytoplasm and membrane and the membrane expression of *α*ENaC exhibited no significant difference (Figures 5(c) and 5(d)).

Obesity-linked insulin resistance is associated with inflammation in adipocytes. Among the different types of proinflammatory cytokines, TNF*α* is the first one identified to connect obesity, inflammation, and insulin resistance. Notably, TNF*α* at both 5 and 10 ng/ml can upregulate p-PPAR*γ* (Figure 5(e)), while it had no effect on *α*ENaC in membrane and AQP2 in the cytoplasm and membrane (Figures 5(f) and 5(g)).

## 4. Discussion

In this study, we determined the reason for the relationship between fluid retention caused by ROS and the expression of AQP2 and *α*ENaC in HEK293 and mIMCD-3 cells. Immunofluorescence experiment reveals that the green fluorescence in the nucleus increased after ROS application compared with that in the control, illustrating that ROS can activate the transcriptional activity of transcription factors and promote their transfer from the cytoplasm to the nucleus. Next, we studied the effects of ROS on p-PPAR*γ* at the protein level in HEK293 and mIMCD-3 cells. As shown in Figures 3(b) and 4(b), in both HEK293 and mIMCD-3 cells, ROS (10 *μ*M) can critically inhibit PPAR*γ* phosphorylation, in terms of both the total level and that in the nucleus; upon coincubation with GW9662, an antagonist of PPAR*γ*, all of these effects disappeared. Taking these findings together, ROS activated PPAR*γ*, leading to the reduction of p-PPAR*γ*.

Against this background, to determine whether the reduction of p-PPAR*γ* could lead to fluid retention, we examined the expression of AQP2 and *α*ENaC proteins, which are related to body fluid homeostasis [10, 11]. The trafficking mechanism of AQP2 was mainly induced by arginine vasopressin (AVP); when AVP increased, the cytoplasmic vesicles and lumen membrane fused, and AQP2 was transferred to the luminal membrane, increasing the permeability to water [12]. Based on these findings, an abnormal mechanism of AQP2 trafficking would affect the number of AQP2 molecules in the luminal membrane [13]. In diabetic model mice in vivo, after feeding on PPAR*γ* agonist, PCR detection results showed that ADH had no significant changes, but AQP2 significantly increased [14]. Our results revealed that ROS (10 *μ*M) can increase the expression of AQP2 in the cytoplasm and facilitate AQP2 vesicles fusing to the cell membrane; in addition, after coincubation with GW9662, these effects were all offset. These findings may be explained by ROS increasing the expression of AQP2 in the collecting duct membrane and increasing water reabsorption, leading to fluid retention.

The regulation of renal sodium (Na^+^) handling is a key determinant of the long-term control of extracellular fluid volume homeostasis. Na^+^ reabsorption is mediated via the amiloride-sensitive epithelial sodium channel (ENaC), which exhibits high selectivity for sodium [15] and is a central requirement for Na^+^ reabsorption across renal epithelia. ENaC expression and translocation to the plasma membrane are tightly regulated by a diverse array of hormonal [16, 17] and physical factors [18]. Our experiments show that ROS (10 *μ*M) remarkably raised the membrane level of *α*ENaC, and thus more and more Na^+^ flowed into the lumen, which accelerated water reabsorption and caused more serious fluid retention. After incubation with GW9662, this effect dissipated.

SR1664 is a novel PPAR*γ* ligand (a nonagonist PPAR*γ* ligand) that blocked the cyclin-dependent kinase 5 (CDK5)-mediated phosphorylation of PPAR*γ*. In vivo experiments show that it can increase insulin sensitivity and does not cause fluid retention [5]. This study focused on the relationship between SR1664 and AQP2/*α*ENaC proteins in vitro, indicating that SR1664 downregulated p-PPAR*γ*, but did not cause any significant changes in AQP2 and *α*ENaC in the membrane, demonstrating that SR1664 increased insulin sensitivity without affecting water and sodium channel protein expression.

Activated PPAR*γ* suppresses the expression of TNF*α* [19, 20], and TNF*α* increases the level of insulin antagonistic hormones by phosphorylating serine residue of the substrates of the insulin receptor, inhibiting tyrosine phosphorylation of this receptor, which in turn limits signal transmission [21]. Furthermore, both ROS and SR1664 are reported to eliminate p-PPAR*γ* upregulated by TNF*α* [22]. Prompted by these findings, we also studied the correlation of phosphorylated PPAR*γ* induced by TNF*α* with AQP2 and *α*ENaC expression. As shown in Figure 5, TNF*α* at both 5 and 10 ng/ml can upregulate p-PPAR*γ*, but the expression of AQP2 and *α*ENaC in the cytoplasm and membrane was not changed by it.

In conclusion, in vitro the fluid retention induced by ROS is closely associated with two major aspects: the first includes the increase of cytoplasmic AQP2 and promotion of AQP2 vesicles to undergo membrane fusion, thereby increasing water reabsorption; the second involves ROS enhancing membrane *α*ENaC expression, thus accelerating Na^+^ reabsorption, which further increases the absorption of water. In contrast, SR1664 and TNF*α* experiments reveal that whether activated PPAR*γ* was up- or downregulated did not affect AQP2 and *α*ENaC expression. From the former evidence, we deduced that in vitro there was little association between the fluid retention induced by ROS and the phosphorylation of PPAR*γ*.
